# Physicochemical, Mechanical, and Structural Properties of Bio-Active Films Based on Biological-Chemical Chitosan, a Novel Ramon (*Brosimum alicastrum*) Starch, and Quercetin

**DOI:** 10.3390/polym14071346

**Published:** 2022-03-26

**Authors:** Soledad Cecilia Pech-Cohuo, Héctor Martín-López, Jorge Uribe-Calderón, Nancy Guadalupe González-Canché, Iván Salgado-Tránsito, Alejandro May-Pat, Juan Carlos Cuevas-Bernardino, Teresa Ayora-Talavera, José Manuel Cervantes-Uc, Neith Pacheco

**Affiliations:** 1 Centro de Investigación y Asistencia en Tecnología y Diseño del Estado de Jalisco CIATEJ, A.C. Subsede Sureste, Parque Científico Tecnológico de Yucatán, Mérida 97302, Yucatán, Mexico; spech_al@ciatej.edu.mx (S.C.P.-C.); hemartin_al@ciatej.edu.mx (H.M.-L.); tayora@ciatej.mx (T.A.-T.); 2 Centro de Investigacion Cientifica de Yucatan, Unidad de Materiales, Mérida 97205, Yucatán, Mexico; jorge.uribe@cicy.mx (J.U.-C.); amay@cicy.mx (A.M.-P.); manceruc@cicy.mx (J.M.C.-U.); 3 Centro de Investigaciones en Óptica, Unidad de Aguascalientes, Prol. Constitución 607, Aguascalientes 20200, Aguascalientes, Mexico; ngonzalc@cio.mx (N.G.G.-C.); isalgadotr@cio.mx (I.S.-T.); 4 CONACYT—Centro de Investigación y Asistencia en Tecnología y Diseño del Estado de Jalisco, Subsede Sureste, Parque Científico Tecnológico de Yucatán, Mérida 97302, Yucatán, Mexico; jcuevas@ciatej.mx

**Keywords:** Ramon starch, biological-chemical chitosan, bioactive films

## Abstract

The properties of biological-chemical chitosan (BCh) films from marine-industrial waste and a non-conventional Ramon starch (RS) (*Brosimum alicastrum*) were investigated. Blended films of BCh/RS were prepared to a volume ratio of 4:1 and 1:4, named (BChRS-80+q, biological-chemical chitosan 80% *v*/*v* and Ramon starch, BChRS-20+q, biological-chemical chitosan 20% *v*/*v* and Ramon starch, both with quercetin), Films from commercial chitosan (CCh) and corn starch (CS), alone or blended (CChCS-80+q, commercial chitosan 80% *v*/*v* and corn starch, CChCS-20+q commercial chitosan 20% *v*/*v* and corn starch, both with quercetin) were also prepared for comparison purposes. Films were investigated for their physicochemical characteristics such as thickness, moisture, swelling, water-vapor permeability, and water solubility. In addition, their mechanical and structural properties were studied using Fourier Transform Infrared Spectroscopy (FTIR), Thermogravimetric analysis (TGA) and Scanning Electron Microscopy (SEM) techniques. Antioxidant activity was evaluated as radical scavenging, and antimicrobial effect was also determined. The BCh and RS films presented similar tensile strength values compared with commercial biopolymers. Only films with chitosan presented antioxidant and antimicrobial activity. The FTIR spectra confirmed the interactions between functional groups of the biopolymers. Although, BChRS-80+q and BChRS-20+q films exhibited poor mechanical performance compared to their commercial counterparts, they showed good thermal stability, and improved antioxidant and antimicrobial activity in the presence of quercetin. BChRS-80+q and BChRS-20+q films have promising applications due to their biological activity and mechanical properties, based on a novel material that has been underutilized (Ramon starch) that does not compete with materials for human feeding and may be used as a coating for food products.

## 1. Introduction

Plastic materials have been widely used in food packaging; however, these are produced from non-biodegradable petrochemical polymers, which increase the risk of environmental pollution and are associated with health effects due to the migration of specific components into food processing or storage [1,2]. The above has attracted interest on active food packaging materials based on biodegradable and biopolymers such as polysaccharides, proteins, and lipids [1,3,4]. Biopolymer-based films can reduce moisture loss, restrict oxygen permeation, and provide physical protection for food [1,5]. These properties are highly related to the biopolymer employed [6].

There is currently an emerging interest in using wastes and by-products from agricultural activities and the food industry to obtain biopolymers [7,8]. Millions of tons of residues cause nutritional, economic, and environmental problems every year [9]. Therefore, the effective use of these wastes and by-products in developing new products could ensure the sustainability of the food industry, reduce environmental pollution, and provide added value to these wastes [9,10]. It is also essential to develop new efficient methods that do not consume a lot of time and energy and do not generate high quantities of residues [11]. Therefore, it is essential to explore alternative resources for obtaining interest compounds for the food industry, for example to elaborate biodegradable films.

Starch is a promising biopolymer for food packaging; it is abundant in nature, renewable, biodegradable, and inexpensive [4]. Several studies report starch isolation processes for unconventional sources, such as huaya seeds (*Melicoccus bijugatus*) [12] and parotta (*Enterolobium cyclocarpum*) [13], which do not participate with human feeding and can be also used as raw materials for material obtentions [13,14]. In addition, starch can also be obtained from Ramon seeds (*Brosimum alicastrum*), a tree found in Southeastern Mexico [15]. Its average production of seeds is 95.5 kg per year [16,17], and each seed possesses 92.57% of starch with an amylose content of 25.36% [14,18]. This plant species is considered underutilized and undervalued [19]; nevertheless, recent studies report the thermoplastic material [19] and adhesives [20] produced from RS.

In general, starch can be processed with existing polymer processing technologies, and starch-based materials have excellent oxygen barrier properties [21]. However, these materials present poor moisture barrier properties, brittleness, and a low tensile strength, limiting their applications [4]. In his sense, starch can be blended with other natural biopolymers to form composite materials to mitigate these disadvantages and obtain additional functional properties [4].

Chitosan is a natural cationic biopolymer obtained from the deacetylation of chitin by chemical or biological methods. The biological method produces chitosan of higher molecular weight and appropriate mechanical properties, contributing to reducing waste generated during chemical deacetylation processes [2,22]. It is the second most abundant polysaccharide after cellulose and is mainly obtained from seafood processing residues [4]. Chitosan has excellent film-forming capacity, antimicrobial activity, and biocompatibility. It has been added to starch matrices from distinct sources, such as, tapioca [23], corn [4], potato [24], wheat [25], cassava [26], and kudzu [27], developing films with improved antimicrobial, mechanical and barrier properties [2,4]. In this sense, different phenolic compounds have also been incorporated into chitosan blends to obtain food packaging films with antimicrobial and antioxidant capacities to increase the shelf life of products and enhance their safety and sensory properties, while preserving food quality [1,4,28].

In recent years, the development of bioactive coatings and films to protect food quality has become widespread, increasing the shelf life of perishable foods, especially those susceptible to oxidative and microbiological decomposition [29,30]. For example, Wang et al. [30] developed edible coatings form nanofibers isolated from whey protein using carvacrol to enhance their antimicrobial properties during food preservation. It is also possible to use the exopolysaccharide of lactic acid bacteria in bioactive films to improve their antioxidant and antimicrobial properties in the food industry [31]. In another study, soy protein emulsion films were developed using different temperatures of thermal pretreatments to improve their food preservation properties [32]. In addition, other soy protein films reinforced with nanofibers from soybean residues or by-products have been developed to increase their food protection properties [33].

Food manufacturers are very interested in using natural antioxidants (such as ascorbic acid, tocopherol, and quercetin) to replace synthetic antioxidants [34,35]. Quercetin is one of the most usual dietary phenolic compounds broadly distributed in medicinal herbs, fruits, vegetables, and red wine [1]. Due to its capability to scavenge free radicals and resist lipid peroxidation, quercetin has a strong antioxidant ability [36]. Therefore, incorporating quercetin into biopolymer-based films can significantly improve the antioxidant capacity of the films.

Quercetin has been successfully incorporated into various distinct films based on one biomaterial: either chitosan or starch films [28,37] or complex blended systems such as chitosan-gelatin [38] and cassava-starch-carboxymethyl cellulose [34,39]. The importance and innovation of this study lies in the use of chitosan obtained from shrimp shell waste through a biological-chemical process, blended with starch from an alternative source that is not used as a human food (Ramon seed), also obtained in an eco-friendly method, and enhancing its bioactive properties with natural compounds for the elaboration of films for food packaging. To the best of our knowledge, there are no publications concerning the formation of biological-chemical chitosan and Ramon starch films. Therefore, the main objective of this work was the evaluation of the physicochemical, mechanical, and structural properties and the antioxidant and antimicrobial capacity of BCh and RS films and their blends compared to those elaborated from commercial CCh and CS, and the effect of quercetin in the improvement of their biological activity, providing information on their performance when applied in the food industry.

## 2. Materials and Methods

Commercial chitosan (CCh; molecular weight 310–375 kDa: degree of deacetylation ≥ 75%; 419419 product number) was purchased from Sigma-Aldrich (Sigma Aldrich, St. Louis, MO, USA). Biological chitin was provided by Keiko Shirai Matsumoto (Unidad de Biotecnología, Universidad Autonoma Metropolitana Unidad Iztapalapa, Ciudad de Mexico, Mexico) and was deacetylated according to Martín-López et al. [40] to obtain a final biological-chemical chitosan (BCh) (≈107 kDa: degree of deacetylation ≈ 79%). Ramon starch (75% amylopectin, 25% amylose) was produced from *Brosimum alicastrum* seeds according to methodology reported by Pech-Cohuo et al. [41] using deionized water. Corn starch (73% amylopectin, 27% amylose) and quercetin (purity ≥ 98%) were purchased from Sigma-Aldrich (Sigma Aldrich, St. Louis, MO, USA).

2,2′-azino-bis-3-ethylbenzothiazoline-6-sulphonic acid (ABTS), 2,2-diphenyl-1-picrylhydrazyl (DPPH), glycerol, sodium bromide, 6-hydroxy-2,5,7,8-tetramethylchroman-2-carboxylic acid (Trolox), and acetic acid was of analytical grade and purchased from Sigma-Aldrich (Sigma Aldrich, St. Louis, MO, USA). Gram-negative *Salmonella typhimurium* (ATCC 14028) and Gram-positive *Staphylococcus aureus* (ATCC 25923) strains were acquired from the internal ceparium of the food safety and traceability laboratory from Centro de Investigacion y Asistencia en Tecnologia y Diseño del Estado de Jalisco, Subsede Sureste. Deionized water was used for all the experiments.

### 2.1. Preparation of Chitosan and Starch Films

Chitosan stock solution was prepared by dissolving BCh and CCh powders (2% *w/v*) in 100 mL of acetic acid solution (1% *v/v*) at 25 °C according to Pacheco et al’s methodology. [2]. Ramon (RS) and corn (CS) starch stock solutions (1% *w/v*) were dispersed in deionized water at 80 °C for 15 min following the methodology of Pacheco et al. [2]; beakers were covered with water-resistant film to minimize water evaporation. Glycerol was added to chitosan and starch stock solutions (at 25% with respect to the total biopolymer mass).

Films were elaborated by the casting method according to Martin-Lopez et al. [40]. The solutions were agitated for 10 min and treated in an ultrasonic bath (Bransonic 3510R-MT, Branson Ultrasonics, Co., Danburi, CT, USA) for 10 min. Then the solutions were placed into Petri dishes (0.3 g/cm^2^) and dried at 50 °C for 48 h. The obtained films were stored using a NaBr-saturated solution at 50% relative humidity (RH) at 25 °C for 48 h [40].

### 2.2. Preparation of Chitosan-Starch-Quercetin-Based Films

Blends solutions (100 mL) were prepared with a volume ratio of chitosan (BCh or CCH)/starch (RS or CS) of 4:1 (BChRS-80+q, CChCS-80+q) and 1:4 (BChRS-20+q, CChCS-20+q).All of which were added with quercetin at a concentration of 5 mg/mL in ethanol, in a proportion of 0.35% with respect to the total biopolymer concentration, glycerol was also added to the blends at 25%. The ratios of chitosan/starch evaluated in this study were chosen based on a preliminary study. Several concentrations of biological-chemical chitosan/Ramon starch were tested, and those that presented the best film formation were chosen. Films of BChRS-80+q, CChCS-80+q, BChRS-20+q and CChCS-20+q were prepared according to the methodology described above.

### 2.3. Films Characterization

#### 2.3.1. Physicochemical Properties: Thickness, Moisture, Water Solubility, Degree of Swelling of the Films, and Water Vapor Permeability (WVP)

A total of ten thickness measurements were taken at distinct positions on the films, using a digimatic micrometer (model MDC-1 PXT, Mitutoyo Manufacturing Co. Ltd., Tokyo, Japan) with a sensitivity of 0.001 mm.

The moisture content of the films was measured as described by Pacheco et al. [2]. Film pieces (2 cm × 2 cm) were weighed (*W*_0_), and then were dried in an oven at 105 °C to reach constant weight (*W*_1_). The moisture content was calculated as follows:

(1) $$Moisture\;content\;\left(\%\right)=\frac{W_{0}-W_{1}}{W_{0}}\times 100$$

The water solubility of the films was determined using film pieces (1.4 cm × 1.4 cm) which were dried at 105 °C and weighted (*w*_1_). The film pieces were immersed in 100 mL beakers with 50 mL of distilled water and sealed, stored at room temperature for 24 h and constant agitation to 120 rpm. Additionally, to reach the final dry mass (*w*_2_) the films were dried out with filter papers (weight before) in an oven at 105 °C for 24 h [42]. Then, the water of the films was analyzed using Equation (2):

(2) $$Film\;solubility=\frac{w_{1}-w_{2}}{w_{1}}\times 100$$

For the determination of swelling degree, pieces (2 cm × 2 cm) were dried in an oven, then soaked in 50 mL beakers with 30 mL of distilled water at 25 °C for a given period. The extra water of highly swollen samples was removed using filter paper and the samples were weighed [43]. Experiments were carried out in triplicate, and the average value was measured as the swelling ratio. The degree of swelling was calculated using Equation (3):

(3) $$Film\;swelling\;degree=\frac{w_{1}-w_{2}}{w_{1}}\times 100$$

In the above formula, *w*_1_ and *w*_2_ are the weights of dry and wet film samples, respectively.

A gravimetrically methodology was used for the determination of the water vapor permeability (WVP) of the films according to the method reported by Pacheco et al. [2] with slight modifications. The films were cut into 5 cm diameter circles and stored at 25 °C and 50% RH for 48 h. Circles of the samples were placed and sealed on the top of a plastic container (4 cm in diameter) with 15 mL distilled water. Parafilm was used to avoid moisture loss through the seals. The samples were then placed into a desiccator with 1 kg of silica gel at 25 °C and weighed every 2 h for 24 h. WVP was estimated using Equation (4):

(4) $$WVP=\frac{\left(dW\right)\left(L\right)}{\left(dt\right)\left(A\right)\left(dP\right)}$$

where, *dW* is the weight loss (g) of the container of the samples, *L* is the film thickness (m), *A* is the area (m^2^), and *dt* is the time (s) under the partial water vapor pressure gradient (*dP*). Samples were evaluated in triplicate.

#### 2.3.2. Mechanical Properties

Tensile strength (TS) and elongation at break (EAB) were determined following the standard method ASTM D882 using a Universal testing machine (model AGS-X, Shimadzu Scientific Instruments, Columbia, MD, USA). Before measurement, film samples (2.5 cm × 10 cm) were stored for three days at 25 °C and 50% relative humidity. The test conditions were initial grip separation and crosshead speed were set to 50 mm and 50 mm/min at 25 °C until break. The stress-strain curve obtained was used to calculate TS (MPa), elastic modulus (MPa) and EAB (%).

#### 2.3.3. Structural Properties

##### Thermogravimetric Analysis

Thermogravimetric analysis (TGA) was performed in a TGA-DSC, simultaneous analyzer NETZSCH (model STA 449 F5, NETZSCH, Selb, Germany). The samples (10 mg each) were heated from 25 °C to 600 °C at a constant heating rate of 10 °C/min into a He atmosphere and a flow rate of 20 mL/min.

##### FTIR Analysis

The FTIR spectra were used to analyze the structural interactions between biopolymers. The FTIR spectra of the films were obtained in the frequency range from 4000 to 400 cm^−1^ at a resolution of 8 cm^−1^ on a spectrometer (Model Nicolet 8700, Thermo Scientific, Madison, WI, USA).

##### Scanning Electron Microscopy (SEM)

Samples were prepared by immersing a piece (5 mm × 5 mm) cut from the film into liquid nitrogen. The film piece was broken into several smaller pieces with a previously cooled razor blade. The films were mounted on carbon sample holders and sputtered with gold. Images of the surface of the samples were observed using a Scanning Electron Microscope (Model JSM-6360, JEOL, Tokyo, Japan) at an accelerating voltage of 10 kV and magnified 1000×.

#### 2.3.4. Bioactive Evaluation

##### DPPH Radical Scavenging Activity (RSA_DPPH_)

The antioxidant activity of the films was evaluated using the DPPH method according to Pacheco et al. [2] with slight modifications. In total, 25 mg of each film was added into 3 mL of distilled water. Then, 2.8 mL of each film extracted solution was mixed with 0.2 mL of DPPH methanolic solution (1 mM) at room temperature in the dark for 30 min; the mixture was then shaken and incubated. Using a UV-vis spectrophotometer (BioMate 3S, Thermo Fisher Scientific Inc., Waltham, MA, USA), the absorbance was measured at 517 nm. DPPH antioxidant activity was described as Trolox equivalent concentration (μg_TEAC_/mL) and radical scavenging activity was calculated as follows:

(5) $$RSA_{DPPH}\left(\%\right)=\frac{A_{control}-A_{sample}}{A_{control}}\times 100$$

where *A_control_* = absorbance of DPPH solution without the addition of the film and *A_sample_* = absorbance of the sample solution. Trolox standard curves were conducted. Films elaborated with the biopolymers individually (BCh, CCh, RS, CS) were used as controls.

##### ABTS+ Scavenging Activity (RSA_ABTS_)

The ABTS assay was also used to determine the antioxidant activity of the film samples according to the method described by Pacheco et al. [2]. The antioxidant activity was described as Trolox equivalent concentration (μg_TEAC_/mL) and *RSA* was calculated as follows:

(6) $$RSA_{ABTS}\left(\%\right)=\frac{A_{control}-A_{sample}}{A_{control}}\times 100$$

where *A_control_* = absorbance of ABTS solution without the antioxidant sample and *A_sample_* = absorbance of sample solution. Trolox standard curves were conducted. Films elaborated with the biopolymers individually (BCh, CCh, RS, CS) were used as controls.

##### Antibacterial Activity

The films’ antimicrobial activity was evaluated with two strains: *Staphylococcus aureus*, Gram-positive bacterial and *Salmonella* *typhimurium*, Gram-negative bacterial [44]. The method of Perumal et al. [45] and Martin-López et al. [40] was used with slight modifications. A suspension of each strain was prepared and adjusted to 10^6^ UFC/mL by comparison with standard solution McFarland 0.5 [44]. A 100 μL aliquot of each strain solution was extended on a dish surface with Mueller Hinton culture medium. Then, a piece of each film (diameter = 1.8 cm) was placed on the center of the dishes. According to Martin-Lopez [46], the diameter used improves the visibility of the inhibition zone and facilitates the measurement of the increment area. The dishes were incubated at 37 °C for 24 h. Microbial growth on the samples and controls was corroborated using staining with *p*-iodonitrotetrazolium chloride solution (0.25 mg/mL). Inhibition diameter was expressed as diameter (mm) of clear zone around the disk and percentage of area increase according to Equation (7) [47]:

(7) $$\%area\;increase=\frac{A_{final}-A_{onset}}{A_{onset}}\times 100\;$$

Sterile paper disks previously immersed in sterile saline solution were used as a negative control while sterile paper disks soaked with 10 μL of ciproflaxin solution (25 μg/mL) were used as a positive control.

### 2.4. Statistical Analysis

An Analyses of Variance (ANOVA) and a Tukey’s means comparison test was applied to the data. Statistical significance was stablished at *p* < 0.05, using Statgraphics Centurion XVI software (Statistical Graphics Corp., Warrenton, VA, USA, Manugistics, Inc., Cambridge, MA, USA).

## 3. Results

### 3.1. Physicochemical, Mechanical and Bioactive Evaluation of Chitosan and Starch Films

#### 3.1.1. Thickness, Moisture, Solubility, Swelling Index, and Water Vapor WVP

The thickness, moisture, solubility, and swelling results of BCh, CCh, RS and CS are presented in Table 1. Thickness and moisture values for BCh and CCh films were not significantly different (0.15 mm of thickness and ≈19% of moisture for both samples). RS and CS films presented the same thickness values, nevertheless RS film showed a higher moisture content (32.31%) compared to the CS film (15.39%). BCh and CCh films show similar solubility values (≈25 and 27%, respectively), while for the RS film (≈53%) a slightly higher solubility value is observed than for the CS film (≈50%). Ramon and corn starch films presented the highest values of swelling degree (112 and 240%, respectively); BCh and CCh film for water vapor permeation (6.75 and 7.80 × 10^−9^ (g m^−1^ s^−1^ Pa^−1^), respectively)., but there were no significant differences among them in both determinations. The Section 4 describes the implications of these values on the films for each property in more detail.

#### 3.1.2. Mechanical Properties

The tensile strength, elongation at break, and elastic modulus are also shown in Table 1. BCh (2.43 MPa), CCh (3.05 MPa), RS (2.49 MPa), and CS (3.20 MPa) films showed similar tensile strength values. Elongation at break values were higher for BCh (25.29%) and RS (17.43%) films, whereas the lowest value was for the corn starch film (7.93%). For the elastic modulus, the films with the highest values were RS (50.54 MPa) and CCh (25.83 MPa), and the film with the lowest value was CS (1.22 MPa). Concerning tensile strength, it would be indistinct to use chitosan and starch of non-conventional or commercial origin; however, for EAB and elastic modulus, significant differences are observed between the samples.

#### 3.1.3. Antioxidant Activity

The RS and CS films showed no antioxidant activity for both assays. Concerning DPPH and ABTS radical scavenging activity, the results showed no statistically significant differences between the chitosan films evaluated (Table 1), which makes viable the use of both materials for the elaboration of bioactive films. The antioxidant activity presented by chitosan is mainly due to the amino groups in its structure that interact with the free radicals of DPPH and ABTS solutions [2]. However, the inherent antioxidant capacity of chitosan can be enhanced by the use of polyphenols such as quercetin [38].

#### 3.1.4. Antimicrobial Activity

Table 1 shows the antimicrobial activity values expressed as inhibition percentage of the evaluated strains. RS and CS films showed no antimicrobial activity when evaluated with both strains. The negative control showed microbial growth for both strains under the paper disk, while the ciprofloxacin solution in the positive control diffused, causing no microbial growth on the agar. Martin-Lopez et al. [40] reported similar observations. The BCh and CCh films presented similar values (without significant statistical differences) in the inhibitory microbial percentage for both strains, which allows using these materials indistinctly for this purpose. The antimicrobial behavior shown by chitosan films is related to how protonated groups can interact with microbial cells; three mechanisms have been proposed to explain this behavior [48,49], which were described in detail in the Section 4. Some studies report that some polyphenols, such as quercetin, can also enhance the inhibition of microorganism growth on chitosan films. Therefore, adding this polyphenol to the films could increase their bioactivity [28,38].

### 3.2. Structural Characteristics

#### 3.2.1. TGA

**Figure 1:**
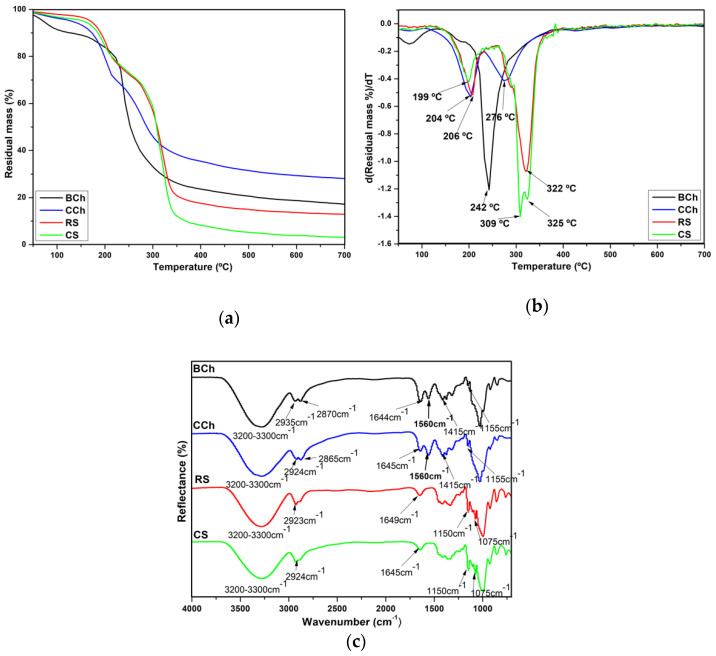
TGA (**a**) and DTGA (**b**) curves and (**c**) FTIR spectra for BCh, CCh, RS and CS films.

The TGA and its derivative (DTGA) curves, as well as, the maximum decomposition temperature (T_dmax_) are shown in Figure 1a,b. The residual mass percentage values for BCh, CCh, RS and CS are as follows: 17%, 27%, 14% and 3%, respectively. CS presented a lower residual mass percentage value because it contains fewer impurities than RS. It has been reported that ashes in starches are related to proteins, phosphate groups, and minerals such as calcium and magnesium [50]. The T_dmax_ values were determined from the minimum temperatures of the peaks in the TGA curve derivatives. The CS film presented three T_dmax_ (199, 309 and 325 °C), while BCh (185 and 242 °C), RS (206 and 322 °C), and CCh (204 and 276 °C) films presented two T_dmax_. The T_dmax_ of the RS and CS films are similar to those reported for Ramon starch [41] and corn starch [51] biopolymers, respectively. Based on previous results for BCh and RS films, blends of these biopolymers may result in films with improved thermal properties.

#### 3.2.2. FTIR Analysis

The spectra of RS and CS films are presented in Figure 1c; the characteristic peaks which occurred at 1649 cm^−1^ and 1645 cm^−1^, respectively, were due to the presence of bound water and the broad band which appeared from around 3280 cm^−1^ was due to hydrogen-bonded hydroxyl groups. The peaks at 1150 cm^−1^ were attributed to the stretching vibration of C–O in C–O–H groups and the peak at 1075 cm^−1^ was attributed to the stretching vibration of C–O in C–O–C groups. For the chitosan films there was a broad band ranging from around 3200 to 3300 cm^−1^ which was attributed to the stretching vibration of N–H and hydrogen-bonded hydroxyl groups, and the peak at 2780 cm^−1^ was attributed to C–H stretching. The peaks at 1649 cm^−1^ and 1645 cm^−1^, for the BCh and CCh film, respectively, were associated with C=O stretching (amide-I). These also relate to water bound to the biopolymer; however, the signal may be overlapping. The peaks at 1560 and 1415 cm^−1^ were attributed to N–H and O–H bending, respectively.

#### 3.2.3. SEM

**Figure 2:**
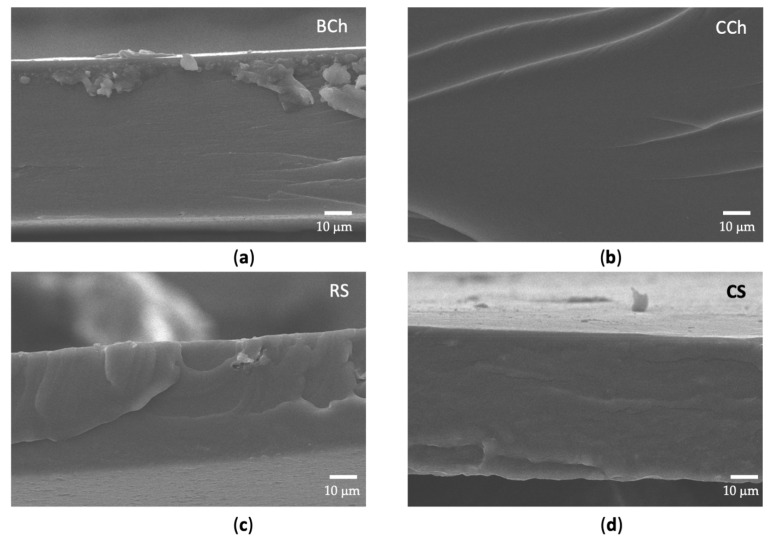
SEM micrographs (**a**) BCh, (**b**) CCh, (**c**) RS and (**d**) CS films.

SEM micrographs are presented in Figure 2a–d for BCh, CCh, RS, and CS. The surface of the biological and commercial chitosan films is uniform and smooth. Regarding the starch films, it is observed that Ramon starch presents a rougher surface than that of corn starch, which is smooth and uniform. The failure mode of chitosan films appears brittle whereas it is slightly ductile for starch films. These observations can be related to the mechanical behavior of the films.

### 3.3. Physicochemical, Mechanical, and Bioactive Evaluation of Blended Chitosan/Starch Films

#### 3.3.1. Thickness, Moisture, Solubility, Swelling Index, and Water Vapor Permeability (WVP)

**Figure 3:**
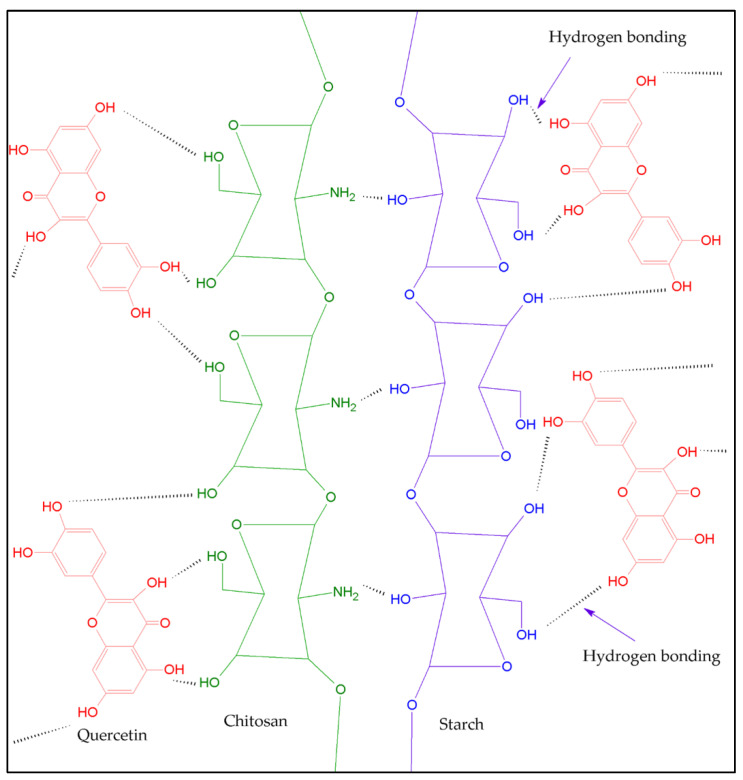
Scheme of hydrogen bonding interaction between chitosan (green structure), starch (blue structure), and quercetin (molecule in red color).

Figure 3 shows chitosan, starch, and quercetin structures and electrostatic interaction through bonding hydrogen between them in the blends. Yadav et al. [38] reported similar interactions for quercetin/starch in blends with gelatin and chitosan.

Table 2 shows the thickness, moisture, solubility, and swelling results of chitosan-starch blend films. BChRS-80+q (0.15 mm) and CChCS-80+q (0.13 mm) films have similar thickness values, while BChRS-20+q (0.06 mm) and CChCS-20+q (0.04 mm) films had the lowest values. As for moisture percentage, films with higher chitosan content presented high values in comparison to films with higher starch content. The BChRS-80+q and BChRS-20+q films showed solubilities between ≈52 and 57%, while CChCS-80+q and CChCS-20+q films show ≈52% and 36% solubilities, indicating that the proportion of commercial chitosan influenced this property. For the degree of swelling, the films with the highest proportion of starch presented the highest values of this property. The RSBCh-80+q, RSBCh-20+q, CChCS-80+q, and CChCS-20+q films presented lower swelling values compared to those obtained for films of an individual biopolymer. Concerning WVP values, the highest values were observed in BChRS-80+q and CChCS-80+q films. On the other hand, films containing a higher proportion of starch show the lowest water vapor permeation values.

#### 3.3.2. Mechanical Properties

The tensile strength, elongation at break and elastic modulus are shown in Table 2. BChRS-80+q and BChRS-20+q films presented the lowest tensile strength values with 0.56 MPa and 0.68 MPa, respectively. In contrast, the values for CChCS-80+q and CChCS-20+q films showed the highest mechanical property values with 3.61 MPa and 7.47 MPa, respectively. Elongation at break values were higher for CChCS-80+q and CChCS-20+q films (20.10 and 34.50%, respectively), whereas the lowest value was found for the RSBCh-80+q film (4.80%). For the elastic modulus, the films with the highest values were CChCS-20+q (40.79 MPa) and the film with the lowest value was BChRS-80+q (6.72 MPa). The mechanical properties presented for BChRS-80+q and BChRS-20+q films may be due to the low interaction between the molecular chains of BCh and RS.

#### 3.3.3. Antioxidant Activity

Concerning DPPH radical scavenging activity, the results showed no statistically significant differences among the films evaluated; however, a slight increase in the values of this measurement was observed for the films containing quercetin compared to the films elaborated from individual biopolymers. The film results presented no statistically significant differences in the ABTS radical assay, making it feasible to use these materials indistinctly due to their bioactive characteristics. However, for the ABTS test, higher values were observed for films from the blends than those made from the biopolymers separately. Thus, quercetin improved the antioxidant capacity in the films of the blends.

#### 3.3.4. Antimicrobial Activity

Table 2 shows the antimicrobial activity values. For the *S. Aureus* strain, higher inhibitory activity values can be observed for the films with higher chitosan content. In the case of the *S. Typhimurium* strain, BChRS-80+q and CChCS-80+q films show similar values without significant statistical differences. These values are higher compared to BChRS-20+q and CChCS-20+q films. The film that showed a little inhibitory activity for both strains was CChCS-20+q. However, the antimicrobial activity for the blend films showed a positive effect with quercetin as higher values of percentage inhibition were obtained for both strains in contrast to the films of each biopolymer.

### 3.4. Structural Characteristics of Blended Chitosan and Starch Films

#### 3.4.1. TGA

**Figure 4:**
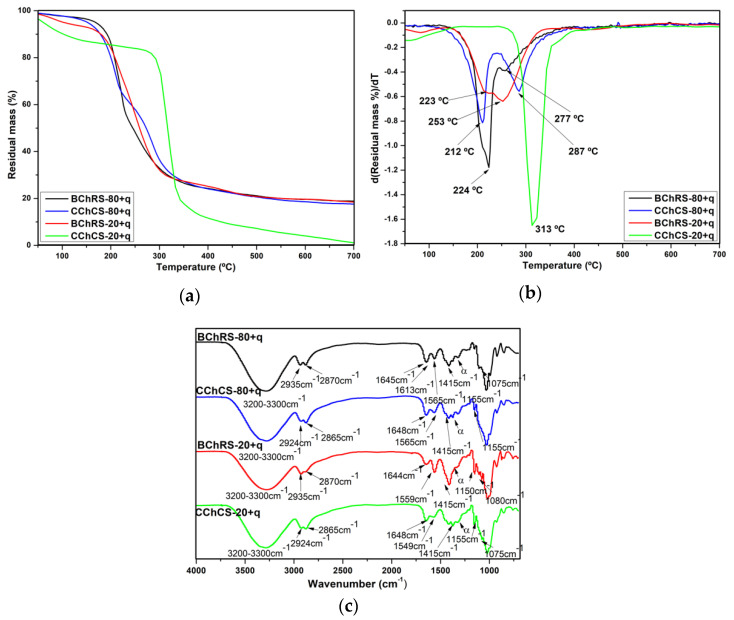
TGA (**a**) and DTGA (**b**) curves and (**c**) FTIR spectra for BChRS-80+q, CChCS-80+q, BChRS-20+q, and CChCS-20+q (The peak α corresponds to 1320 cm^−1^).

TGA and DTGA curves, as well as the maximum decomposition temperature (T_dmax_), are shown in Figure 4a,b. The residual mass percentage values for BChRS-80+q, CChCS-80+q, BChRS-20+q and CChCS-80+q are as follows: 18%, 18%, 19% and 2%, respectively. CChCS-80+q presented a lower residual mass percentage value possibly due to the lower amount of impurities in CS. The BChRS-80+q (224 and 277 °C), BChRS-20+q (223 and 253 °C) and CChCS-80+q (212 and 287 °C) films presented two T_dmax_, while CChCS-20+q (313 °C) presented only one T_dmax_. BChRS-80+q and CChRS+80+q presented similar behaviors in the TGA and DTGA curves; however, the peaks with the two T_dmax_ are more defined in the CChCS-80+q film. In the case of CChCS-20+q, a well-defined peak was observed at 313 °C on the DTG curve instead of the two less pronounced peaks (223 °C and 253 °C).

#### 3.4.2. FTIR Analysis

Chitosan/starch composites spectra presented the peaks already described in Section 3.2.2 for chitosan, and starch biopolymers were also observed (Figure 4c). However, in the spectra, changes in the wavenumbers and intensity of characteristic peaks were observed, according to other reports [4,38]. In the spectra for BChRS-80+q, BChRS-20+q, CChCS-8+q, and CChCS-8+q, peaks at 1613 cm^−1^ and 1320 cm^−1^ (α in Figure 4c) are related to quercetin. These were attributed to the C=C stretching vibration of the phenyl ring and C–O stretching vibration [38,52,53], respectively. However, quercetin peaks are overlapped with the signals of the biopolymers.

#### 3.4.3. SEM

**Figure 5:**
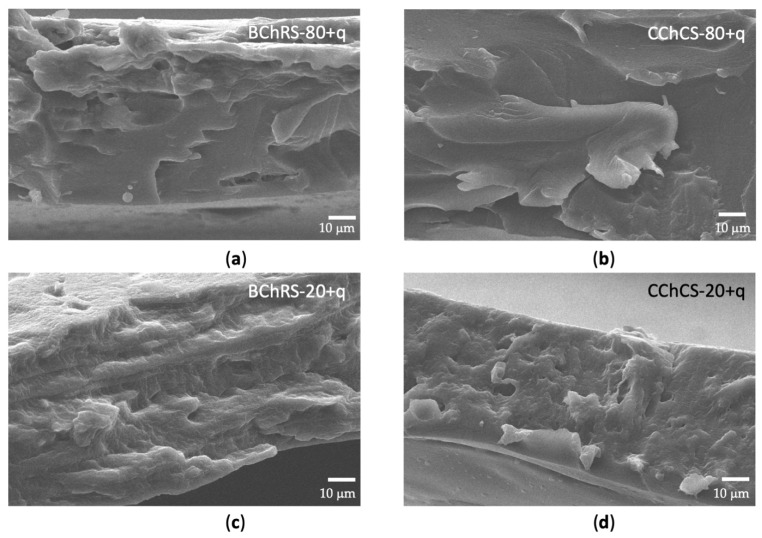
SEM micrographs of (**a**) BChRS-80+q, (**b**) CChCS-80+q, (**c**) BChRS-20+q and (**d**) CChCS-20+q films.

SEM micrographs are presented in Figure 5a–d. The BChRS-80+q, BChRS-20+q, CChCS-80+q, and CChCS-20+q films appear with a rough surface and holes associated with the separation of quercetin crystals. Films with higher starch content show greater roughness and streaks and a more significant number of voids. The failure mode is related to ductile behavior. The surfaces observed in the micrographs can be related to the mechanical behavior of the films.

## 4. Discussion

Determining chitosan/starch-based films’ solubility in water is essential for food packaging film [38]. The solubility behavior of films has been observed in films in other studies of mixtures of commercial chitosan and corn starch and is attributed to the fact that starch is highly soluble in water [4]. In this sense starch films added with chitosan decrease solubility; additionally, the solubility in these films may also be influenced by quercetin content. This polyphenol exhibits an amphipathic behavior due to the phenyl rings that form the hydrophobic part of the molecule and the hydroxyl groups of the polar portion; however, quercetin solubility in water is deficient [28].

The swelling index indicates the capacity of a film to retain water in its matrix, which is related to the interaction of the surrounding water molecules with the hydrophilic groups (e.g., carboxyl or hydroxyl groups) of the film [2]. Similar studies were performed and state that the swelling of these biopolymer blends is mainly due to water diffusion and polymeric relaxation [2,38,54]. Compared with the values obtained for films elaborated from individual biopolymers, the lower swelling values of blend films are attributed to the hydrophobic nature of quercetin. Due to the interaction between quercetin with the chitosan molecules by hydrophobicity or hydrogen bonding, the swelling values decrease, which results in the reduction of the water molecules by the starch molecules [28]. Furthermore, this also results in less exposure of the polar side-chain groups to the water molecules [28].

Water vapor permeation (WVP) is a property that indicates the capacity of the films to interact with water and encourage preservation against the dehydration process or re-hydration of the food [4], preventing or at least reducing the exchange of moisture between the packaged products and the surrounding environment [28]. The highest WVP values corresponding to BCh, BChRS-80+q, CCh, and CChCS-80+q films may be related to the more significant number of free hydroxyl groups and the hydrophilic glycerol. Free hydroxyl groups improved the interactions with surrounding water molecules, encouraging water vapor transmission through the films. Moreover, glycerol favors the reduction of inter-molecular hydrogen and the increase in the distance between the molecular chains, which might encourage the adsorption-desorption of surrounding water molecules [4,55]. Similar observations have been reported; this trend can be explained by the higher hydrophilicity (NH3^+^ groups) of films with higher chitosan content [4,56].

A biodegradable film must possess specific mechanical properties to maintain its integrity and barrier ability during handling, shipping, and storage [2,4]. The properties of CCh and CS resulted in a notable increase in the tensile strength of CChCS-80+q and CChCS-20+q films. The improved tensile strength can be related to enhanced interaction between NH_3_^+^ of the commercial chitosan backbone and OH− of the corn starch by forming intermolecular hydrogen bonds. In the acetic acid solution, the amino groups (NH_2_) of chitosan were protonated to NH_3_^+^. It can also be attributed to the difference in molecular weights between commercial chitosan (310–375 kDa) and biochemical chitosan (107 kDa). In addition, several aforementioned studies discussed the fact that the ordered crystalline structures of starch molecules collapsed during the gelatinization process, and OH- groups exposed formed hydrogen bonds with NH_3_^+^ of the CCh readily [4,56]. In the case of BChRS-80+q and BChRS-20+q, it had reported that the molecular interaction between starch and chitosan chains could reinforce the tensile strength of the films, nevertheless, here the tensile strength values were low. The above was attributed to the concentration of NH_3_^+^ groups exceeding a critical value. The formation of homogeneous composites in BChRS-80+q and BChRS-20+q apparently resulted in weak boundary interactions and lower tensile strength [4]. These can indicate the mechanical properties of the BChRS-80+q and BChRS-20+q films depending on the higher miscibility of the two biopolymers as well as the critical concentration of the components in the blends [4]. Furthermore, the type of chitosan and polyphenol concentration had different effects on the tensile strength [2,57], because of the formation of the micro holes and cavities in the films with the incorporation of quercetin which is observable from the SEM image (Figure 5c,d) as previously reported [38]. The elastic modulus, or Young’s modulus, describes the stiffness of the film, and a lower modulus elastic suggests a more flexible material. The incorporation of chitosan has been reported to significantly reduce the modulus elastic and result in more flexible films [4]. As shown in Table 2, modulus elastic was reduced in the presence of chitosan, which is consistent with several studies related to the reduction of crystallinity of starch in the blend films [4,58]. Since mechanical properties are related to crystalline changes, analyses on this property are suggested. Values of elongation at break determines when the film breaks under tensile strength [4]. This property is expressed as the percentage change from the original length of the sample when it is stretched [4]. The films with the highest starch content (BChRS-20+a and CChCs-20+q) had the highest elongation at break values. The increased flexibility of the films could be due to the interaction between glycerol molecules, acting as aplasticizer, and the biopolymer chains which facilitated the chain sliding and thus enhanced the overall flexibility and chain motion [4,59]. In general, the mechanical properties of the films formed by the biological-chemical chitosan and Ramon starch were observed to be decreased when the composites were formed. The interaction between BCh and RS is possibly not adequate for film-forming. It is suggested to analyze the surface interactions and consider coupling agents or to modify the biopolymers superficially, as well as the evaluation of coating materials due to their characteristics. Nevertheless rheological measurements are needed. Due to their low mechanical properties, blends of these biopolymers could also be considered for use as food coatings, which could deliver the polyphenol into the product. The films formed with commercial chitosan and corn starch showed improved mechanical properties, with the CChCS-20+q film showing the highest values.

TGA was performed to evaluate the thermal stability of the chitosan, starch, and composite chitosan-starch- blend films for their application in the food and pharmaceutical industry; during their preparation, processing, or consumption, the films may be subjected to heat processes [60]. The well-defined peak in the DTGA curve of the starches is related to a reaction mechanism for the degradation of starch biopolymers (amylose and amylopectin) [18,41]. The biological and commercial chitosan films showed significant weight loss from 200 to 500 °C, which is attributed to the pyrolytic decomposition of the saccharide ring dehydration and polysaccharide depolymerization [61,62]. The biological chitosan film showed lower thermal stability than the commercial chitosan film. It has been found that chitosan prepared from different sources (fish, shrimp, and crab) has low thermal stability compared with commercial chitosan [62]. This can also be attributed to the differences in molecular weights between commercial and biological-chemical chitosan origin. In a TGA-FTIR study, the complex gas mixture during chitosan degradation was mainly composed of CO_2_, NH_3_, COCH_3_COOH, and CH_4_ [61]. The release of H_2_O, NH_3_, CO_2_, CO, and CH_3_COOH was observed in the temperature range 250–450 °C, which was related to the pyrolytic degradation of chitosan. NH_3_ was liberated at a lower temperature than the other gases. The release of CH_4_ took place in the range of 450–750 °C, suggesting a modification of the material by releasing methane and forming a graphite-like structure through a hydrogenation mechanism [61]. The CChCS-80+q and CChCS-20+q films showed higher thermal stability than those made with RS and BCh. This is possible due to the influence of the commercial chitosan and its higher thermal stability [62]. In general, the following stages were observed in the TGA curves, which are attributed to Td_max_, between 72 and 186 °C is related to the evaporation of water absorbed by starch, chitosan, and glycerol, as well as evaporation of low molecular weight compounds [60]. The fragmenting of starch, chitosan and quercetin occurs at around 250–350 °C [60,63] and in a third stage at temperatures above 400 °C [64,65]. These last two stages correspond to complex processes, including the dehydration of saccharide rings, depolymerization, and the decomposition of acetylated and deacetylated units of a biopolymer [64].

According to ATR-FTIR, results suggested some interactions between the hydroxyl groups of starch and the amino groups of chitosan. The intensity of the peak located at ~1645 cm^−1^ of starch decreased in the BChRS-20+q and CChCS-20+q films, and the peak at ~1560 cm^−1^ related to chitosan was higher for BChRS-80+q and CChCS-80+q, which is related to changes in the concentration of biopolymers. The characteristic peaks of quercetin are 1613 cm^−1^ and 1320 cm^−1^; however, these were overlapped by the signals from the chitosan and starch peaks. Possibly due to the concentration of quercetin added to the films. Furthermore, slight shifts in the position of some bands were observed. For example, the peak at 1560 cm^−1^ in the BCh film was located at 1565 cm^−1^ in BChRS-80+q. For the blend films with commercial chitosan and corn starch, the peak at 1560 cm^−1^ presented in the CCh film shifted to 1565 and 1549 cm^−1^ in the CChCS-80+q and CChCs-20+q films, respectively. Ren et al. [4] related these changes to the interaction of inter- and intra- molecular hydrogen bonding between chitosan and starch. Similar behavior was observed for films with commercial chitosan and corn starch. This behavior could be related to the effects of the chitosan and starch concentration on the kind and number of polymer-solvent, polymer-polymer, and polymer-plasticizer interactions. The above depends on the properties of the biopolymers [4].

The microstructure and polymer compatibility at the cross-sections of the composite films was observed using SEM [4]. The uniform and smooth surface of the BCh and CCh films suggests an ordered matrix in both cases, which has been observed previously [28,66]. Furthermore, it has been observed that Ramon starch presents a rougher surface than corn starch. The above is probably related to elongation at break values of RS. The BChRS-80+q and CChCS-80+q films also appear with a rough surface and holes, associated with the separation of quercetin crystals. A fibrous surface with a nodular needle-like shape related to the formation of quercetin crystals that tends to aggregate in a tightly packed arrangement has been observed on chitosan-quercetin films [28]. This is because of the low solubility of quercetin in water, which may explain the formation of crystals and their presence on the surface [28,67]. The BChRS-20+q and CChCS-20+q films presented much more roughness and voids than BChRS-80+q and CChCS-80+q, possibly due to the higher starch content and the voids by separation of quercetin. However, the BChRS-20+q composite, in addition to those mentioned above, presents a much more striated surface associated with its low tensile strength. Ren et al. [4] reported that for films with 21% chitosan in starch, rougher surfaces are observed compared to films with 81% chitosan.

Food products are susceptible to oxidation processes during production and storage, which produce a sequence of discouraging sensory properties (e.g., changes in color, taste, texture, and odor), leading to a decrease in quality and economic losses [28,68]. The incorporation of antioxidants in packaging materials to control oxidation can contribute to preserve the quality of food products [28]. The determination of antioxidant activity was performed by DPPH and ABTS assay methods. The RS and CS films showed no antioxidant activity when evaluated by the two assays as expected. Concerning DPPH radical scavenging activity, in the case of BCh and CCh films, the antioxidant activity is caused by the amino groups in the chitosan structure which react with free radicals resulting in the formation of macromolecular free radicals and highly stable ammonium groups [2]. It has been reported that in chitosan/gelatin films with a quercetin/starch complex [38], enhanced antioxidant activity was observed upon addition of quercetin as it is one of the most effective antioxidants due to its structure with many functional groups [1]. However, it has also been reported that increasing the concentration of quercetin and TBHQ (quercetin and tertiary butylhydroquinone) had no effect on the DPPH scavenging activity of Cassava starch/gelatin and Cassava starch/carboxymethyl cellulose films [34,39]. In the food industry, ABTS is used to measure antioxidant activity, a blue-green compound that decolorizes in the presence of an antioxidant [28]. The antioxidant behavior of chitosan is attributed to the fact that the free radicals can react with remaining free amino groups (-NH_2_) of chitosan and form ammonium groups (-NH_3_^+^) towards the absorption of hydrogen ions from the solution [28,69]. As in the DPP assay, quercetin-added films showed higher antioxidant capacity than chitosan films. The structural characteristics of quercetin have been identified as determinants in its free radical scavenging and antioxidant activity [37]. The hydroxyl groups in its structure may be dehydrogenated, deprotonated, or oxidized [37]. The capacity of the phenolic groups of polyphenol to contribute hydrogens to stabilize free radicals was maintained after addition into the composites films [28].

Determining the antimicrobial activity of bioactive films is essential to ensure that packaged food products retain their shelf life, quality, and safety [70]. The antimicrobial characteristics of chitosan are related to a more significant number of protonated groups accessible to interact with microbial cells [48,49]. Three mechanisms have been proposed to describe the antimicrobial activity of this biopolymer: 1) chitosan functional groups interact with microbial membrane phospholipids causing loss of cellular content; 2) chitosan activates important chelating elements causing a deficiency of nutrients; and 3) by penetrating the cell wall and interacting with DNA, it affects protein synthesis [48,71]. In the case of BChRS-20+q and CChCS-20+q films where the lowest antimicrobial capacity was observed, it is possibly due to the higher amount of Ramon and corn starches since both materials showed no inhibitory capacity. Similar behavior was observed for composites with a higher amount of starch [25]. Regarding BChRS-80+q and CChCS-80+q films, unlike biological and commercial chitosan films, the increase in antimicrobial capacity is due to the addition of polyphenol. Quercetin has been reported to exhibit antibacterial activity related to inhibiting nucleic acid synthesis (inhibition of DNA gyrase), in addition to increased bacterial inner membrane permeability, and membrane dissipation potential [28]. The electrochemical gradient of protons across the membrane is essential for the bacterium to conserve the capacity to synthesize ATP and the functions of transport and movement through the membrane [28,36]. Quercetin appears to affect the gradients as well as cell viability [28].

The behavior of biochemical chitosan films and chitosan/Ramon starch composites can be used as a natural antibacterial agent. Therefore, preventing the growth of bacteria in food and restraining contamination by pathogenic bacteria, thus extending the shelf life and improving the safety of food products.

## 5. Conclusions

This work presents the development of bioactive films containing BCh from marine-industrial waste and a non-conventional RS from an underutilized seed native to the southeastern region of Mexico. They were successfully prepared by a simple and eco-friendly method, such as solution casting. In addition, the antioxidant characteristics of the blend of these biopolymers were enhanced by adding quercetin. The BCh and RS films exhibited mechanical properties similar to their commercial counterparts (commercial chitosan and corn starch). The values for BChRS-80+q and BChRS-20+q films were lower than for CChCS-80+q and CChCS-20+q, which may be due to inhomogeneous integration. The use of coupling agents is suggested to generate chemical-type bonds that may be necessary to improve the mechanical properties to allow it to be used as a film; nevertheless, the characteristics presented may be used as food coatings that could deliver the quercetin compound to the product. The above was also observed in SEM micrographs by finding more striated surfaces with voids, indicating that quercetin interaction is weak, and it can be easily released to the product. BCh, RS, BChRS-80+q, and BChRS-20+q films presented similar thermal properties to their commercial counterparts. Due to their thermal behavior, they are an alternative which can be used in packaging films for the food industry contributing to the utilization of waste with the application of an underutilized starch source that does not compete as human feed. Ramon starch film did not show antioxidant or antimicrobial activity, while the biological-chemical chitosan presented both activities similar to commercial chitosan. Biological-chemical chitosan presents itself as an option to produce materials with antioxidant and antimicrobial capacity. At the same time, BChRS-80+q, and BChRS-20+q films showed enhanced antioxidant and antimicrobial activity due to quercetin and the chitosan amount. The results of BChRS-80+q and BChRS-20+q films suggest that developing antibacterial and antioxidant biocomposites is possible from BCh and RS. Thus, these materials may be helpful to increase the shelf life of food products.

## Figures and Tables

**Table 1:** Physicochemical and mechanical properties, antioxidant, and antimicrobial activities of BCh, CCh, RS and CS films.

Film	BCh ^i^	CCh ^ii^	RS ^iii^	CS ^iv^
Thickness (mm)	0.14 ± 0.002 ^a^	0.15 ± 0.009 ^a^	0.06 ± 0.006 ^b^	0.06 ± 0.002 ^b^
Moisture (%)	19.13 ± 1.78 ^b^	18.71 ± 1.74 ^b^	32.31 ± 1.23 ^a^	15.39 ± 0.22 ^c^
Solubilty (%)	24.68 ± 3.97 ^b^	27.74 ± 1.76 ^b^	52.47 ± 5.53 ^a^	49.53 ± 041 ^a^
Swelling (%)	32.00 ± 0.22 ^c^	103.50 ± 0.84 ^b^	111.90 ± 5.92 ^b^	240.44 ± 18.45 ^a^
WVP × 10^−9^ (g m^−1^ s^−1^ Pa^−1^)	6.75 ± 0.11 ^a^	7.80 ± 1.69 ^a^	2.26 ± 0.38 ^b^	2.48 ± 0.31 ^b^
Tensile Strength (MPa)	2.43 ± 0.08 ^a^	3.05 ± 0.74 ^a^	2.49 ± 0.12 ^a^	3.20 ± 0.45 ^a^
Elongation at break (%)	25.29 ± 0.11 ^a^	18.01 ± 0.41 ^b^	17.43 ± 0.38 ^b^	7.93 ± 0.95 ^c^
Elastic modulus (MPa)	11.35 ± 0.07 ^c^	25.83 ± 0.77 ^b^	50.54 ± 3.37 ^a^	1.22 ± 0.05 ^d^
DPPH (μg_TEAC_/mL)	2.63 ± 0.31 ^a^	2.83 ± 0.15 ^a^	NP	NP
ABTS (μg_TEAC_/mL)	6.06 ± 0.67 ^a^	6.69 ± 1.20 ^a^	NP	NP
Inhibition (%) for gram possitive, *S. aureus*	33.62 ± 5.45 ^a^	29.86 ± 2.45 ^a^	NP	NP
Inhibition (%) for gram negative, *S. typhimurium*	28.64 ± 0.65 ^a^	27.31 ± 2.30 ^a^	NP	NP

Data are means ± standard deviations. ^a,b,c,d^ Different letters in the same row indicate significant difference between samples (*p* ≤ 0.05). NP: Not presented. (i) BCh = Biological-chemical chitosan (2% *w/v*); (ii) CCh = Commercial chitosan (2% *w/v*); (iii) RS = Ramon starch (1% *w/v*); (iv) CS = Corn starch (1% *w/v*).

**Table 2:** Physicochemical and mechanical properties, antioxidant, and antimicrobial activities of BChRS-80+q, CChCS-80+q, BChRS-20+q and CChCS-20+q.

Film	BChRS-80+q ^i^	CChCS-80+q ^ii^	BChRS-20+q ^iii^	CChCS-20+q ^iv^
Thickness (mm)	0.15 ± 0.008 ^a^	0.13 ± 0.009 ^a^	0.07 ± 0.005 ^b^	0.04 ± 0.002 ^c^
Moisture (%)	36.45 ± 1.58 ^b^	42.84 ± 0.26 ^a^	17.25 ± 1.09 ^d^	21.85 ± 1.97 ^c^
Solubilty (%)	56.47 ± 6.62 ^a^	51.71 ± 0.31 ^a^	52.06 ± 4.27 ^a^	36.44 ± 3.76 ^b^
Swelling (%)	81.24 ± 3.91 ^b^	11.56 ± 1.59 ^c^	121.69 ± 6.59 ^a^	70.75 ± 6.17 ^b^
WVP × 10^−9^ (g m^−1^ s^−1^ Pa^−1^)	6.76 ± 1.57 ^a^	6.00 ± 1.36 ^ab^	3.43 ± 0.45 ^ab^	2.14 ± 0.02 ^b^
Tensile Strength (MPa)	0.56 ± 0.03 ^c^	3.61 ± 0.29 ^b^	0.68 ± 0.09 ^c^	7.47 ± 0.05 ^a^
Elongation at break (%)	4.80 ± 0.35 ^d^	20.10 ± 0.83 ^b^	9.14 ± 0.68 ^c^	34.50 ± 0.18 ^a^
Elastic modulus (MPa)	9.86 ± 0.70 ^c^	16.97 ± 0.57 ^b^	6.72 ± 0.09 ^b^	40.78 ± 2.15 ^a^
DPPH (μg_TEAC_/mL)	3.70 ± 0.29 ^a^	3.60 ± 0.33 ^a^	3.21 ± 0.20 ^a^	3.31 ± 0.02 ^a^
ABTS (μg_TEAC_/mL)	14.06 ± 1.89 ^a^	20.23 ± 2.32 ^a^	18.81 ± 2.15 ^a^	19.48 ± 5.32 ^a^
Inhibition % for gram possitive, *S. aureus*	41.56 ± 3.81 ^a^	37.97 ± 3.82 ^a^	19.41 ± 1.83 ^b^	14.30 ± 0.56 ^b^
Inhibition (%) for gram negative, *S. typhimurium*	44.07 ± 5.73 ^a^	38.47 ± 3.28 ^a^	17.29 ± 2.30 ^b^	10.03 ± 0.56 ^b^

Data are means ± standard deviations. ^a,b,c,d^ Different letters in the same row indicate significant difference between samples (*p* ≤ 0.05). (i) BChRS-80+q = Biological-chemical chitosan/Ramon starch in volume ratio 4:1, with quercetin (3.5 mg/g_TB_); (ii) CChCS-80+q = Commercial chitosan/Corn starch in volume ratio 4:1, with quercetin (3.5 mg/g_TB_); (iii) BChRS-20+q = Biological-chemical chitosan/Ramon starch in volume ratio 1:4, with quercetin (3.5 mg/g_TB_); (iv) CChCS-20+q = Commercial chitosan/Corn starch in volume ratio 1:4, with quercetin (3.5 mg/g_TB_). TB = Total biopolymer concentration (% *w/v*).

## Data Availability

Not data availability.
